# *Lactobacillus casei* combined with *Lactobacillus reuteri* alleviate pancreatic cancer by inhibiting TLR4 to promote macrophage M1 polarization and regulate gut microbial homeostasis

**DOI:** 10.1186/s12885-023-11557-z

**Published:** 2023-10-30

**Authors:** Zemin Zhu, Bo Yi, Zikai Tang, Xun Chen, Ming Li, Tao Xu, Zhijian Zhao, Caixi Tang

**Affiliations:** 1 Department of Hepatobiliary and Pancreatic Surgery, Zhuzhou Central Hospital, Zhuzhou, China; 2 Department of Trauma Center, Zhuzhou Central Hospital, Zhuzhou, China

**Keywords:** *Lactobacillus casei*, *Lactobacillus reuteri*, TLR4, Macrophage polarization, Gut microbial homeostasis

## Abstract

**Background:**

Pancreatic cancer is a highly lethal disease with no effective treatments. *Lactobacillus casei* (*L. casei*) and *Lactobacillus reuteri* (*L. reuteri*) exhibited therapeutic effects on several cancers, but their roles in pancreatic cancer are unknown. This study aims to explore how *L. casei* & *L. reuteri* influence pancreatic cancer and the underlying mechanisms.

**Methods:**

Pancreatic cancer cells were treated with *L. casei* & *L. reuteri* and co-cultured with macrophages in a transwell system in vitro. Pancreatic cancer xenograft model was established and *L. casei* & *L. reuteri* was used to treat mice in vivo. MTT, CCK-8 assay or immunohistochemical staining were used to determine the proliferation of pancreatic cancer cells or tumor tissues. Transwell assay was applied to test the migration and invasion of pancreatic cells. RT-qPCR was utilized to assess TLR4 and MyD88 expressions in pancreatic cells or tumor tissues. WB, immunofluorescence staining, or flow cytometry was used to evaluate the M1/M2 polarization of macrophages. Besides, the composition of gut microbiota of tumor-bearing mice was determined by 16 S rRNA sequencing, and ultra-high performance liquid chromatography-mass spectrometry (UPLC-MS) untargeted metabolomics was used to evaluate the metabolic profiles of feces.

**Results:**

*L. casei* & *L. reuteri* inhibited the proliferation, migration, invasion of pancreatic cancer cells and pancreatic cancer cell-induced M2 polarization of macrophages by suppressing TLR4. Meanwhile, *L. casei* & *L. reuteri* repressed pancreatic cancer growth and promoted M1 macrophage polarization. Besides, *L. casei* & *L. reuteri* reduced fecal *Alloprevotella* and increased fecal azelate and glutamate in nude mice, while TLR4 inhibitor TAK-242 increased *Clostridia UCG-014*, azelate, uridine, methionine sulfoxide, oxypurinol, and decreased glyceryl monoester in the feces of pancreatic tumor-bearing mice. Fecal oxypurinol and glyceryl monoester levels were positively or negatively associated with gut *Clostridia UCG-014* abundance, respectively.

**Conclusion:**

*L. casei* & *L. reuteri* alleviate pancreatic cancer by inhibiting TLR4 to promote macrophage M1 polarization and regulate gut microbial homeostasis.

**Supplementary Information:**

The online version contains supplementary material available at 10.1186/s12885-023-11557-z.

## Introduction

Pancreatic cancer is a highly lethal disease with insidious onset, difficulty in early diagnosis, and rapid progression [1]. Current therapeutic options for pancreatic cancer mainly comprise surgery, systemic chemotherapy, radiotherapy, and so on. However, the effect of these treatment modalities is limited, and the 5-year overall survival rate of pancreatic cancer is still < 10% over the past decades [2]. In recent years, the morbidity and mortality of pancreatic cancer have been on the rise, posing growing threats to human health [3]. Therefore, there is an urgent need for the development of novel therapeutic approaches for pancreatic cancer.

The important roles of tumor microenvironment (TME) in the development of pancreatic cancer have received much attention in recent years [4]. TME contains cellular components such as immune cells, fibroblasts, adipocytes, endothelial cells, and non-cellular components such as cytokines and extracellular matrix [5]. As one of the most abundant cells in the TME, macrophages are critical regulators of pancreatic cancer progression. With high plasticity and heterogeneity, macrophages can polarize into M1 or M2 phenotype in response to the stimulation of the local microenvironment [6]. M1 macrophages exert anti-tumor effects by secreting pro-inflammatory factors such as interleukin 1β (IL-1β), IL-6, and tumor necrosis factor-α (TNF-α), while M2 macrophages promote tumor growth and immune escape by expressing anti-inflammatory or growth factors such as IL-10, transforming growth factor-β (TGF-β), and vascular endothelial growth factor (VEGF) [7]. Studies have shown that high density of stromal M2 macrophages are strongly associated with poor prognosis of pancreatic cancer patients [8]. Hence, promoting the polarization of macrophages into M1 phenotype could be a potential solution for the treatment of pancreatic cancer.

Gut microbiota, the most abundant microbial community in the body, is involved in a variety of essential physiological functions such as digestion, metabolism, immune regulation, and intestinal defense [9]. The composition and diversity of gut microbiota are influenced by host genetics, diet, drugs and other factors, closely associated with the development of various diseases including cancer [10]. Gut microbiota dysbiosis was observed in pancreatic cancer patients [11, 12]. Altering gut microbiota composition and diversity could inhibit the progression of pancreatic cancer [13–16]. Besides, gut microbiota dysbiosis also affected the effectiveness of chemotherapy, radiotherapy, and immunotherapy for pancreatic cancer [17]. Thus, gut microbiota played crucial roles in pancreatic carcinogenesis and served as a potential therapeutic target for pancreatic cancer.

Probiotics are live microorganisms that can benefit the host when they are administered [18]. Probiotics exert beneficial effects on the host through a variety of mechanisms, including regulation of gut microbiota, improvement of intestinal barrier function, modulation of immune system and neurotransmitter production, inhibition of pathogen replication and so on [19]. Probiotics have been shown to have beneficial effects on cancer prevention and treatment in clinical and preclinical studies [20]. Lactic acid bacteria (LAB) are a group of non-spore-forming, Gram-positive bacteria that ferment sugars to lactic acid. Some LAB strains exhibit probiotic properties, such as *Lactobacillus casei* (*L. casei*) and *Lactobacillus reuteri* (*L. reuteri*). *L. casei* and *L. reuteri* have displayed therapeutic potentials in acute and chronic kidney injury [21], rheumatoid arthritis [22], antibiotic-associated diarrhea [23], osteoporosis [24]. In addition, studies have proven that *L. casei* and *L. reuteri* inhibited the progression of tumors such as liver cancer [25] and colorectal cancer [26, 27], but the roles of *L. casei* and *L. reuteri* in pancreatic cancer are not fully understood.

In this study, we explored the regulatory effects of *L. casei* and *L. reuteri* on macrophage polarization in vitro, followed by assessing the effects of *L. casei* and *L. reuteri* on pancreatic cancer and gut microbiota in vivo. We hope that our study could provide new approaches to the treatment of pancreatic cancer.

## Methods

### Cell culture and treatments

The human pancreatic cancer cell lines (MIA PaCa-2, Panc-1, AsPC-1, BxPC-3) and human monocytic cell lines (THP-1) were purchased from Procell (Wuhan, China). MIA PaCa-2/Panc-1 cells and AsPC-1/BxPC-3/THP-1 cells were cultured in DMEM (#PM150210, Procell, China) and RPMI-1640 (#PM150110, Procell, China), respectively. For MIA PaCa-2 cells, the culture medium was supplemented with 10% FBS (#164210-500, Procell, China), 2.5% horse serum (#164215-100, Procell, China) and 1% penicillin/streptomycin (#PB180120, Procell, China). For Panc-1 cells, AsPC-1 cells and BxPC-3 cells, the culture medium was supplemented with 10%FBS and 1% penicillin/streptomycin. For THP-1 cells, the culture medium was replenished with 10% FBS, 0.05 mM β-mercaptoethanol (#PB180633, Procell, China) and 1% penicillin/streptomycin. To induce differentiation into macrophages, THP-1 cells were treated with 150 nM phorbol 12-myristate 13-acetate (PMA, #AWH0222a, Abiowell, China) for 24 h. All cells were maintained in a humid 5% CO_2_ incubator at 37 °C.

*L. casei* (ATCC 39392) and *L. reuteri* (ATCC 23272) were purchased from the American Type Culture Collection. *L. casei* and *L. reuteri* were grown in Mann–Rogosa–Sharpe Agar (MRS) broth (#TY1885, REBio, China) at 37 °C. When the bacterial growth enters the stationary phase, *L. casei* and *L. reuteri* were collected by centrifugation (3000 g, 10 min), washed with PBS, and resuspended in corresponding culture media of pancreatic cancer cells. For the *Lactobacillus* treatment group, pancreatic cancer cells were treated with 1.64 × 10^7^ CFU/mL *L. casei* and 1.64 × 10^7^ CFU/mL *L. reuteri* and cultured for 24 h.

As for the BxPC-3 cells and macrophages co-culture experiment, a transwell system (#3413, Corning, USA) was introduced. After being treated with or without *L. casei* and *L. reuteri* (*L. casei* & *L. reuteri*) or 50 ng/mL lipopolysaccharide (LPS, #AWH0796a, Abiowell, China) for 24 h, BxPC-3 cells were harvested and seeded in the lower chambers, while macrophages were cultured in the upper chambers. After 24 h, BxPC-3 cells and macrophages were harvested for further detection.

### MTT assay

Cells were seeded in 96-well plates at a density of 5 × 10^3^ cells/well (100 μL/well). After *Lactobacillus* treatment, 5 mg/mL MTT (10 μL/well, #AWC0118b, Abiowell, China) was added to each well and cells were cultured in a 37 °C incubator containing 5% CO_2_ for 4 h. Then the supernatant was discarded after centrifugation, and 150 μL/well of DMSO (#AWC0147a, Abiowell, China) was added to the cells. After vibrating for 10 min, plates were put in a microplate reader and the OD value was assessed at 490 nm.

### Reverse-transcription quantitative PCR (RT-qPCR)

RNA was purified from tissues or cells using TRIzol reagent (#15,596,026, ThermoFisher, USA). cDNA was then synthesized from RNA by using commercial cDNA synthesis kits (#CW2569, Cwbiotech, China). Quantitative PCR was performed on a PCR instrument (#PIKOREAL96, ThermoFisher, USA) using UltraSYBR Mixture (#CW2601, Cwbiotech, China). All data were normalized to β-actin and the sequences of RT-qPCR primers (Tsingke Biotech, China) were listed in Table 1.

**Table 1:** The primers used for RT-qPCR analysis

Gene	Primer sequences
TLR4	forward 5’-CTTTATCCAACCAGGTGCAT-3’
	reverse 5’-TTCTAAACCAGCCAGACCTT-3’
MyD88	forward 5’-CCATGGCTGCAGGAGGTC-3’
	reverse 5’-CAGTTGCCGGATCTCCAAGT-3’
β-actin	forward 5’-ACCCTGAAGTACCCCATCGAG-3’
	reverse 5’-AGCACAGCCTGGATAGCAAC-3’

### Cell counting kit-8 (CCK-8) assay

Cells were inoculated into 96-well plates of 5 × 10^3^ cells/well (100 μL/well). After different treatments, cells were stimulated with CCK-8 solution (10 μL/well, #NU679, Dojindo, Japan) and then cultured in a 37 °C incubator containing 5% CO_2_ for 4 h. Finally, the OD value of cells was determined at 450 nm by a microplate reader.

### Transwell migration/invasion assay

For transwell migration assay, equal numbers of *Lactobacillus*-treated or control BxPC-3 cells were harvested and seeded in the upper chambers (#33318035, Corning, USA) with 100 μL serum-free medium, while the lower chambers were loaded with 500 μL of medium containing 10% FBS. After culturing for 48 h, cells in the lower chambers were fixed with 4% paraformaldehyde (#N1012, New Cell & Molecular Biotech, China) and stained with crystal violet (#C8470, Solarbio, China). After observing and picturing by a microscope, cells were then treated with 10% acetic acid for decoloration and their OD value at 550 nm was detected by a microplate reader. For invasion transwell, Matrigel (#356231, Corning, USA) was applied to precoat the upper chambers before cell seeding, and the rest procedures are equivalent to that of transwell migration assay.

### Flow cytometry

For in vitro experiments, THP-1 macrophages were digested using 0.25% trypsin (#C0201, Beyotime, China) and then washed and resuspend in PBS. In vivo experiments included a tumor dissociation kit (#130-095-929, Miltenyi, Germany) and Percoll (#AWC0193a, Abiowell, China) gradient centrifugation to obtain immune cell suspensions from xenograft tumor tissues as per the manufacturer’s protocol. Next, cell suspensions were incubated with anti-CD11b (#562793, BD Biosciences, USA), along with anti-CD86 (#555660, BD Biosciences, USA) or anti-CD206 (#555954, BD Biosciences, USA) for 30 min at room temperature in the dark. Subsequently, cell suspensions were washed with PBS and centrifuged at 300 g for 5 min. After discarding the supernatant, cells were resuspended in PBS and analyzed using a flow cytometer. The compensation was generated using single stain controls.

### Western blot (WB) analysis

Total proteins were extracted from THP-1 macrophages using RIPA lysis buffer (#AWB0136, Abiowell, China) containing protease inhibitor (#583794, Gentihold, China). BCA assay was used to assess the protein concentration using a commercial kit (#23227, ThermoFisher, USA). Then the proteins were loaded onto SDS-PAGE and electrotransferred onto PVDF membranes (#1620177, Bio-Rad, USA), which were then blocked with 5% skim milk (#AWB0004, Abiowell, China) and incubated overnight with primary antibodies against Arginase-1 (Arg-1, #16001-1-AP, Proteintech, USA), inducible nitric oxide synthase (iNOS, #18985-1-AP, Proteintech, USA) or β-actin (#66009-1-Ig, Proteintech, USA) at 4 °C. The next day, the blots were washed with PBS and probed with horseradish peroxidase (HRP)-conjugated second antibodies (#SA00001-1 and #SA00001-2, Proteintech, USA). After visualizing via ECL WB Substrate (#AWB0005b, Abiowell, China), the blots were imaged and quantified using the Quantity One software.

### Animal model

Four-week-old BALB/C nude mice were bought from Hunan SJA Laboratory Animal Co., Ltd. BxPC-3 cells (5 × 10^6^ cells/100 μL per mouse) were injected subcutaneously into the axilla of the left forelimb of nude mice. Mice in the control group received neither cancer cells nor any treatment. The tumor-bearing nude mice were randomly divided into three groups. Mice in the PC group were gavaged with 200 μL PBS and intraperitoneally injected with 200 μL DMSO per day. Mice in the *Lactobacillus* group were gavaged with *L. casei* (1.64 × 10^7^ CFU/0.02 kg) along with *L. reuteri* (1.64 × 10^7^ CFU/0.02 kg) and intraperitoneal injected with 200 μL DMSO per day. Mice in the TAK-242 group were gavaged with 200 μL PBS and intraperitoneal injected with TAK-242 (5 mg/kg, #HY-11,109, MCE, USA) per day. The tumor volumes and body weight were measured twice a week. Four weeks later, the mice were euthanized by neck dislocation and their tumor tissues were harvested for subsequent assays. The animal experiments in this research were approved by Animal Ethics Committee of Second Xiangya Hospital Central South University (2022724). All methods were carried out in accordance with ARRIVE guidelines and regulations.

### Immunohistochemical (IHC) staining

Paraffin sections of tumor tissues were first prepared using a microtome. After dewaxing and rehydration, the sections were subjected to 0.01 M citrate buffer for antigen retrieval, followed by exposure to 1% periodate for the inactivation of endogenous peroxidase. After rinsing with PBS, the sections were incubated with primary antibody against Ki67 (#ab16667, Abcam, UK) overnight at 4 °C. The next day, the sections were incubated with a secondary antibody (#SA00013-2, Proteintech, USA) for 30 min followed by incubation with DAB working solution (#ZLI-9017, ZSGB-Bio, China) for 5 min at room temperature. After hematoxylin re-staining, gradient alcohol dehydration, and xylene clearing, the sections were sealed with neutral gum and observed under a microscope.

### Immunofluorescence (IF) staining

Paraffin sections were first prepared. After dewaxing and rehydration, the sections were washed with PBS and treated with heat-induced antigen retrieval. After blocking by 3% BSA for 30 min, the sections were then incubated with primary antibody against CD86 (#ab53004, Abcam, UK), CD206 (#ab64693, Abcam, UK), Arg-1 (#ab91279, Abcam, UK), iNOS (#18985-1-AP, Proteintech, USA) overnight at 4 °C. After washing with PBS, the sections were probed with a secondary antibody (#SA00013-2, Proteintech, USA) for 2 h at room temperature in the dark, followed by DAPI (#AWC0293a, Abiowell, China) staining. Finally, the sections were sealed with 10% glycerol and observed under a fluorescent microscope.

### 16 S rRNA sequencing

We commissioned APExBIO (Shanghai, China) to perform 16 S rRNA sequencing on mouse fecal samples. Briefly, the samples were sequenced after total DNA extraction, library construction, quality control, primer design and synthesis, PCR amplification of the V3-V4 region and MiSeq library construction. After species annotation of the sequencing results, the relative abundance of species, alpha diversity, LEfSe analysis and differential species between groups were analyzed.

### Ultra-high performance liquid chromatography-mass spectrometry (UPLC-MS) untargeted metabolomics

Fecal samples of nude mice were collected and dissolved in the extraction solution [methanol: acetonitrile:water = 42:42:16 (v/v/v)]. Samples were then centrifuged at 16,000 g, 4 ºC for 10 min and supernatant (containing metabolites) was retained. Expressions of metabolites were analyzed using a mass spectrometer (#TripleTOF5600+, AB Sciex, USA). After filtration with a 0.22 μm filter, the supernatant samples were separated by an HSS T3 column (100 × 2.1 mm, 1.7 μm; Waters, USA). The single components were then ionized by the ion source of the spectrometer, and the qualitative and quantitative results of the samples were obtained by mass spectrometric data analysis.

### Statistical analysis

Data analysis was performed using GraphPad Prism 7 software. Student’s t-test or one-way analysis of variance (ANOVA) was utilized to determine the statistical significance between two or more groups, respectively. Data were shown as mean ± standard deviation (SD), and *p* < 0.05 were considered statistically significant.

## Results

### ***L. casei*** & ***L. reuteri*** inhibits the proliferation, migration, invasion of pancreatic cancer cells and pancreatic cancer cell-induced M2 polarization of macrophages

**Fig. 1:**
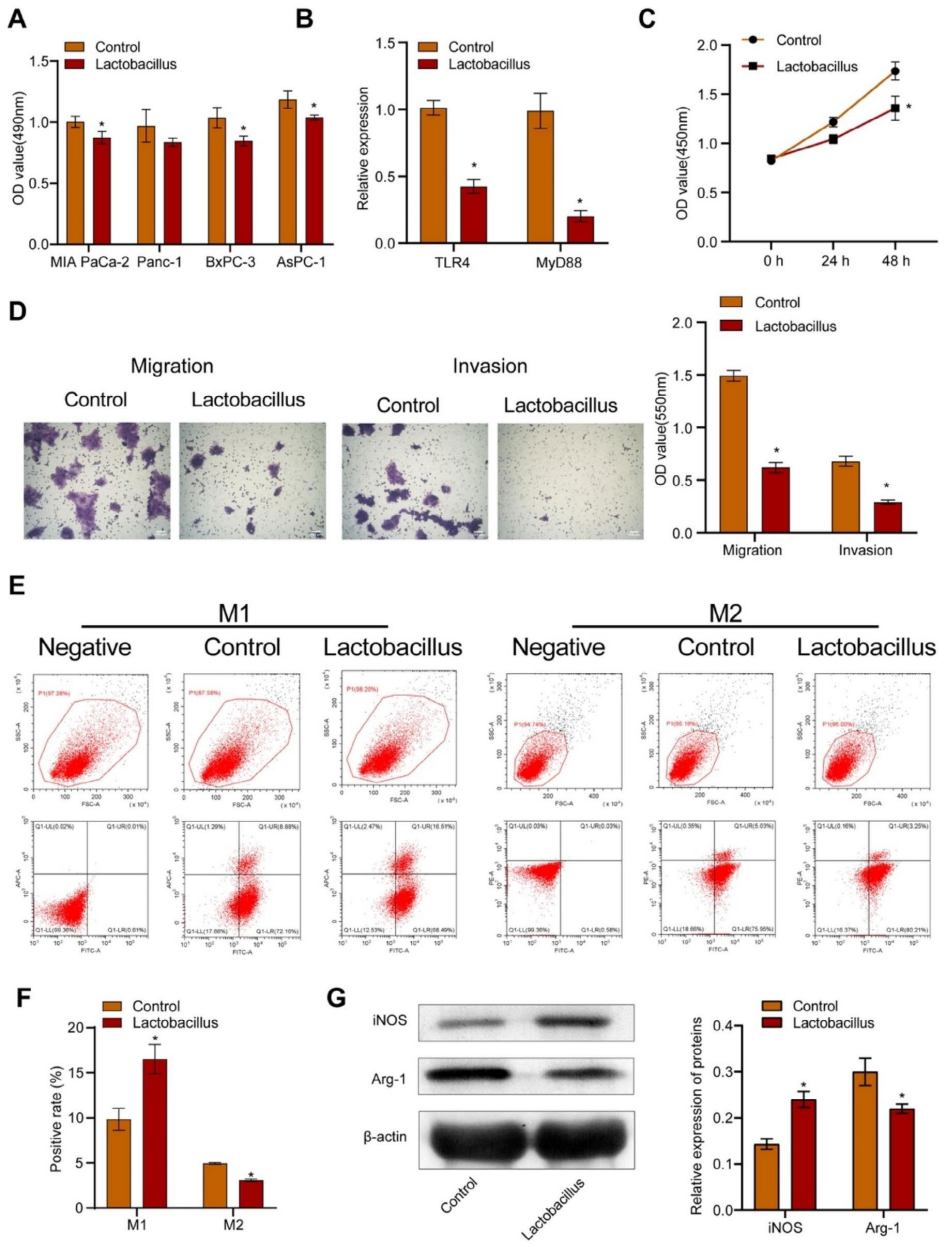
*L. casei* & *L. reuteri* inhibits the proliferation, migration, invasion of pancreatic cancer cells and pancreatic cancer cell-induced M2 polarization of macrophages. (**A**) MTT assay of MIA PaCa-2, Panc-1, BxPC-3, and AsPC-1 cells. (**B**) RT-qPCR analysis of TLR4 and MyD88 expressions in BxPC-3 cells. (**C**) CCK-8 assay of BxPC-3 cells. (**D**) Transwell migration and invasion assay of BxPC-3 cells. (**E**) Representative chart of flow cytometric analysis of CD86^+^ or CD206^+^ macrophages. (**F**) Statistical chart of flow cytometric analysis of CD86^+^ or CD206^+^ macrophages. (**G**) WB analysis of Arg-1 and iNOS in macrophages. Lactobacillus represents cells treated with *L. casei & L. reuteri.* *p < 0.05

We first treated human pancreatic cancer cells (MIA PaCa-2, Panc-1, BxPC-3, and AsPC-1) with *L. casei* & *L. reuteri*. As shown in Fig. 1A, L. *casei* & *L. reuteri* inhibited the proliferation of MIA PaCa-2, Panc-1, BxPC-3, and AsPC-1 cells. Since *L. casei* & *L. reuteri* showed the strongest inhibitory effect on BxPC-3 cell proliferation, we chose BxPC-3 cells as the research objects in the following experiments. We found that *L. casei* & *L. reuteri* downregulated the expressions of Toll-like receptor 4 (TLR4) and myeloid differentiation primary response 88 (MyD88) in BxPC-3 cells (Fig. 1B). Meanwhile, *L. casei* & *L. reuteri* treatment also repressed the proliferation (Fig. 1C), migration and invasion (Fig. 1D) of BxPC-3 cells. To investigate the impact of *L. casei* & *L. reuteri* on macrophages in the TME, we constructed BxPC-3 cells and macrophages transwell co-culture system. Before co-cultured with macrophages, BxPC-3 cells were pre-treated with or without *L. casei* & *L. reuteri* for 24 h. Compared with BxPC-3 cells, *L. casei* & *L. reuteri* treated BxPC-3 cells upregulated M1 markers CD86 (Fig. 1E-F) and iNOS (Fig. 1G), while downregulated M2 markers CD206 (Fig. 1E-F) and Arg-1 (Fig. 1G) in macrophages. These data indicated that *L. casei* & *L. reuteri* inhibited proliferation, migration, invasion of BxPC-3 cells, and attenuated BxPC-3 cell-induced M2 polarization of macrophages.

### ***L. casei*** & ***L. reuteri*** influences pancreatic cancer cells and macrophage polarization by regulating TLR4

**Fig. 2:**
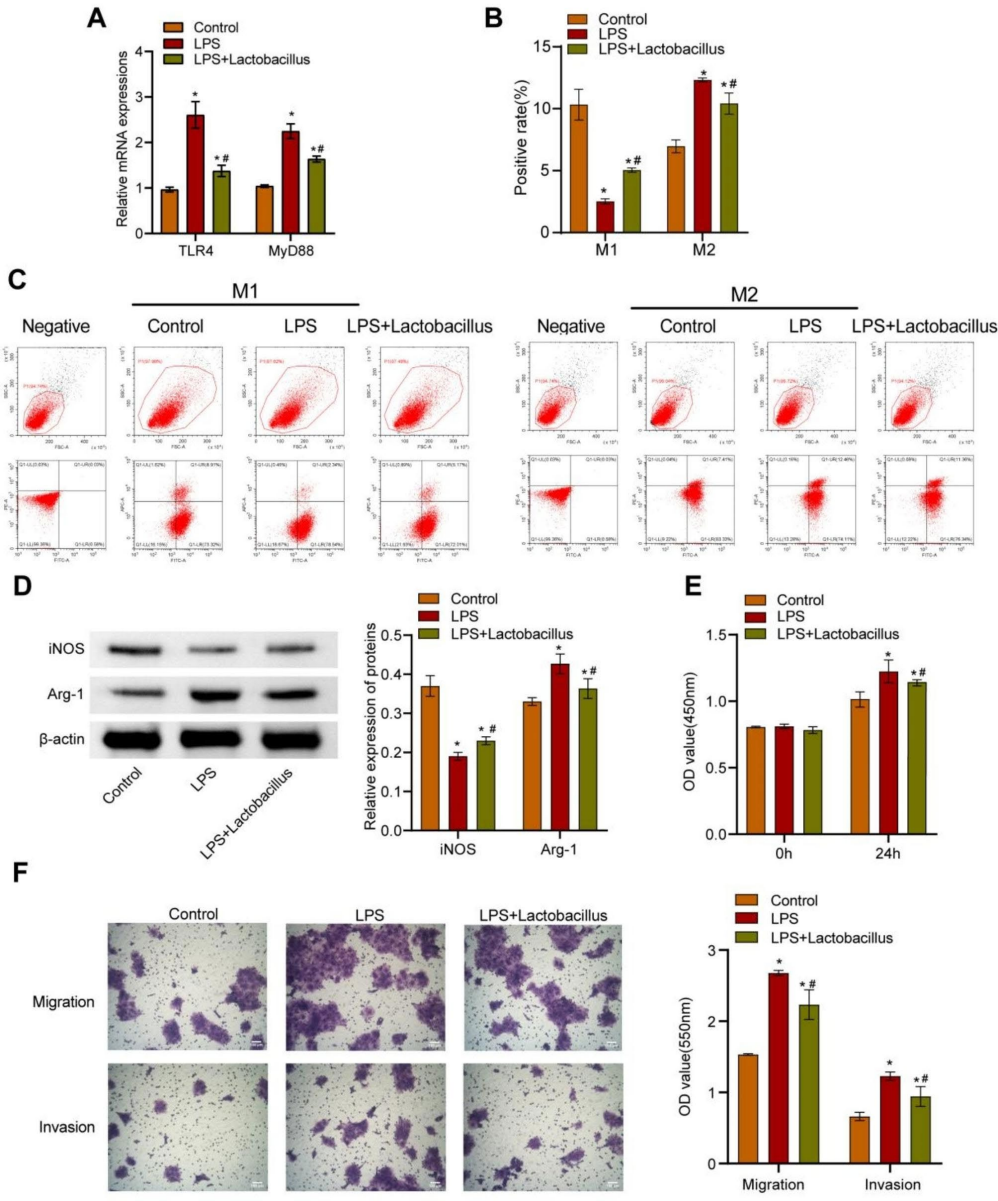
*L. casei* & *L. reuteri* influences pancreatic cancer cells and macrophage polarization by regulating TLR4. (**A**) RT-qPCR analysis of TLR4 and MyD88 expressions in BxPC-3 cells. (**B**) Statistical chart of flow cytometric analysis of CD86^+^ or CD206^+^ macrophages. (**C**) Representative chart of flow cytometric analysis of CD86^+^ or CD206^+^ macrophages. (**D**) WB analysis of Arg-1 and iNOS in macrophages. (**E**) CCK-8 assay of BxPC-3 cells. (**F**) Transwell migration and invasion assay of BxPC-3 cells. *p < 0.05 versus control group, #*p* < 0.05 versus LPS group

To explore whether *L. casei* & *L. reuteri* impacted BxPC-3 cells and macrophage polarization through TLR4, TLR4 agonist LPS was used to stimulate BxPC-3 cells in the following experiments. As present in Fig. 2A, LPS significantly promoted the expressions of TLR4 and its downstream molecule MyD88 in BxPC-3 cells, whereas *L. casei* & *L. reuteri* partly abrogated this effect. Next, we pretreated BxPC-3 cells with or without LPS or *L. casei* & *L. reuteri*, and then we co-cultured BxPC-3 cells with macrophages in a transwell system. Compared with the control group, macrophages in the LPS group showed lower levels of CD86 (Fig. 2B-C), iNOS (Fig. 2D), and higher levels of CD206 (Fig. 2B-C) and Arg-1 (Fig. 2D). However, macrophages in the LPS + *Lactobacillus* group exhibited enhanced CD86 (Fig. 2B-C), and iNOS (Fig. 2D) expressions, and reduced CD206 (Fig. 2B-C), and Arg-1 (Fig. 2D) expressions compared to the LPS group. Besides, the proliferation (Fig. 2E), migration, and invasion (Fig. 2F) of BxPC-3 cells were significantly propelled in the LPS group compared with the control group. Nevertheless, BxPC-3 cells in the LPS + *Lactobacillus* group displayed attenuated proliferation (Fig. 2E), migration, and invasion (Fig. 2F) as compared to the LPS group. These results suggested that *L. casei* & *L. reuteri* influenced pancreatic cancer cells and macrophage polarization by regulating TLR4.

### ***L. casei*** & ***L. reuteri*** represses pancreatic cancer growth and promotes M1 macrophage polarization by inhibiting TLR4

**Fig. 3:**
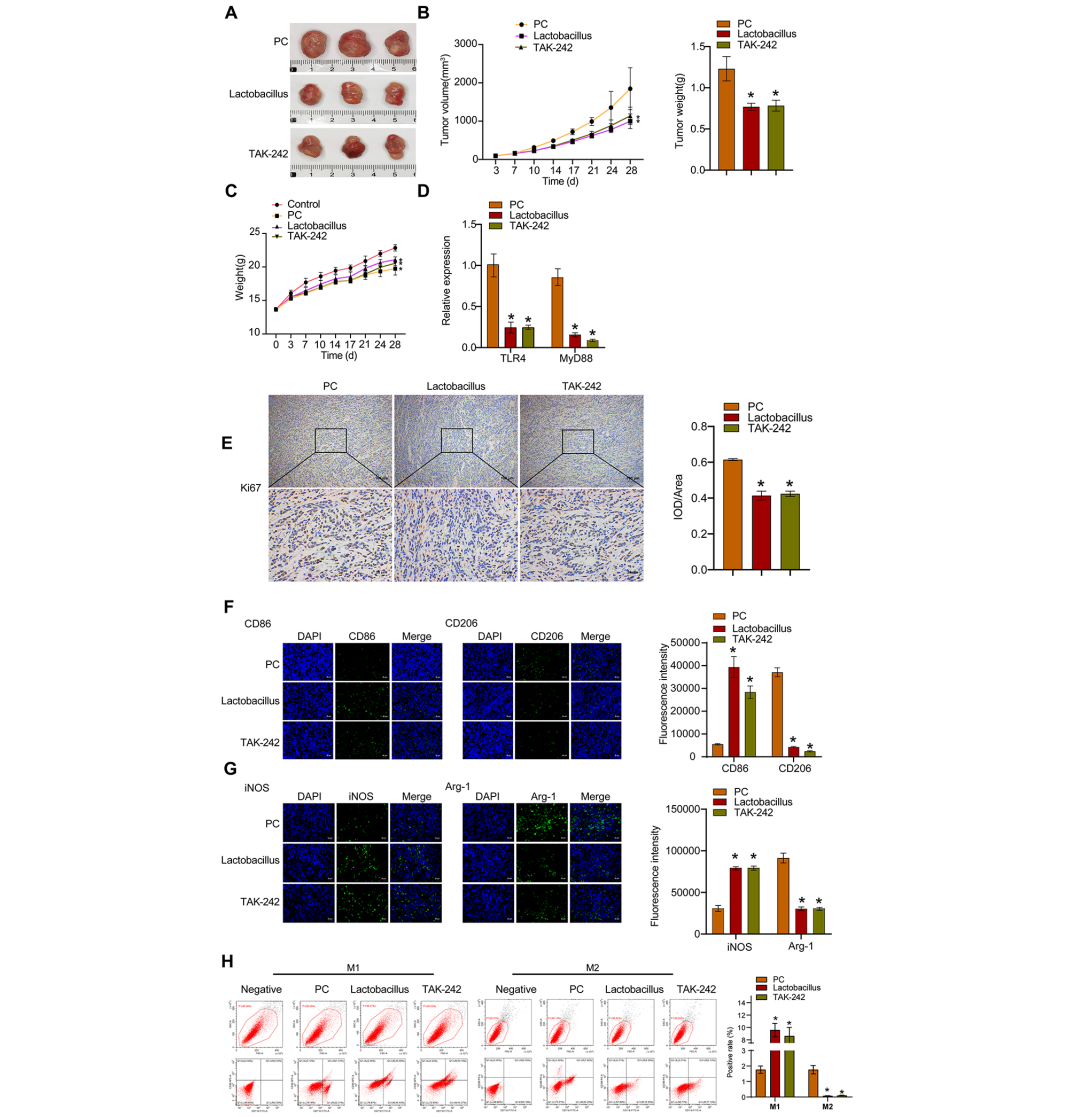
*L. casei* & *L. reuteri* represses pancreatic cancer growth and promotes M1 macrophage polarization by inhibiting TLR4. (**A**) Nude mice were xenografted with BxPC-3 cells (PC group) and treated with *L. casei* & *L. reuteri* (Lactobacillus group) or TAK-242 (TAK-242 group). The tumor sizes are shown. (**B**) The tumor volumes (left) and tumor weights (right) of nude mice. (**C**) Body weights of nude mice. (**D**) RT-qPCR analysis of TLR4 and MyD88 expressions in tumor tissues. (**E**) IHC staining for Ki67 in tumor tissues. Scale bar: 100 μm (upper), 25 μm (lower). (**F**) IF staining for CD86 and CD206 in tumor tissues. (**G**) IF staining for Arg-1 and iNOS in tumor tissues. (**H**) Flow cytometric analysis of CD86^+^ or CD206^+^ macrophages in tumor tissues. *p < 0.05 versus PC group

To explore how *L. casei* & *L. reuteri* influenced the tumorigenicity of pancreatic cancer cells in vivo, the BxPC-3 xenograft model was established in nude mice and *L. casei* & *L. reuteri* or TLR inhibitor TAK-242 were applied to treat mice at the same time. Both *L. casei* & *L. reuteri* and TAK-242 significantly decreased tumor volume (Fig. 3A-B), tumor weight (Fig. 3B), but did not affect the whole body weight of tumor bearing mice (Fig. 3C). Besides, both *L. casei* & *L. reuteri* and TAK-242 profoundly inhibited TLR4, MyD88 (Fig. 3D), and Ki67 (Fig. 3E) expressions in tumor tissues. Furthermore, after *L. casei* & *L. reuteri* or TAK-242 treatment, the expressions of CD86 (Fig. 3F) and iNOS (Fig. 3G) were increased in tumor tissues, whereas the expressions of CD206 (Fig. 3F) and Arg-1 (Fig. 3G) were decreased at the same time. Furthermore, the proportion of M1 macrophages in tumor tissues was markedly increased, while the proportion of M2 macrophages was reduced after *L. casei* & *L. reuteri* or TAK-242 treatment (Fig. 3H). More importantly, *L. casei* & *L. reuteri* and TAK-242 exhibited nearly the same inhibitory effect on tumor growth and macrophage polarization, implying that *L. casei* & *L. reuteri* suppressed pancreatic cancer growth and M1 macrophage polarization by inhibiting TLR4.

### ***L. casei*** & ***L. reuteri*** regulates gut microbial homeostasis by inhibiting TLR4

**Fig. 4:**
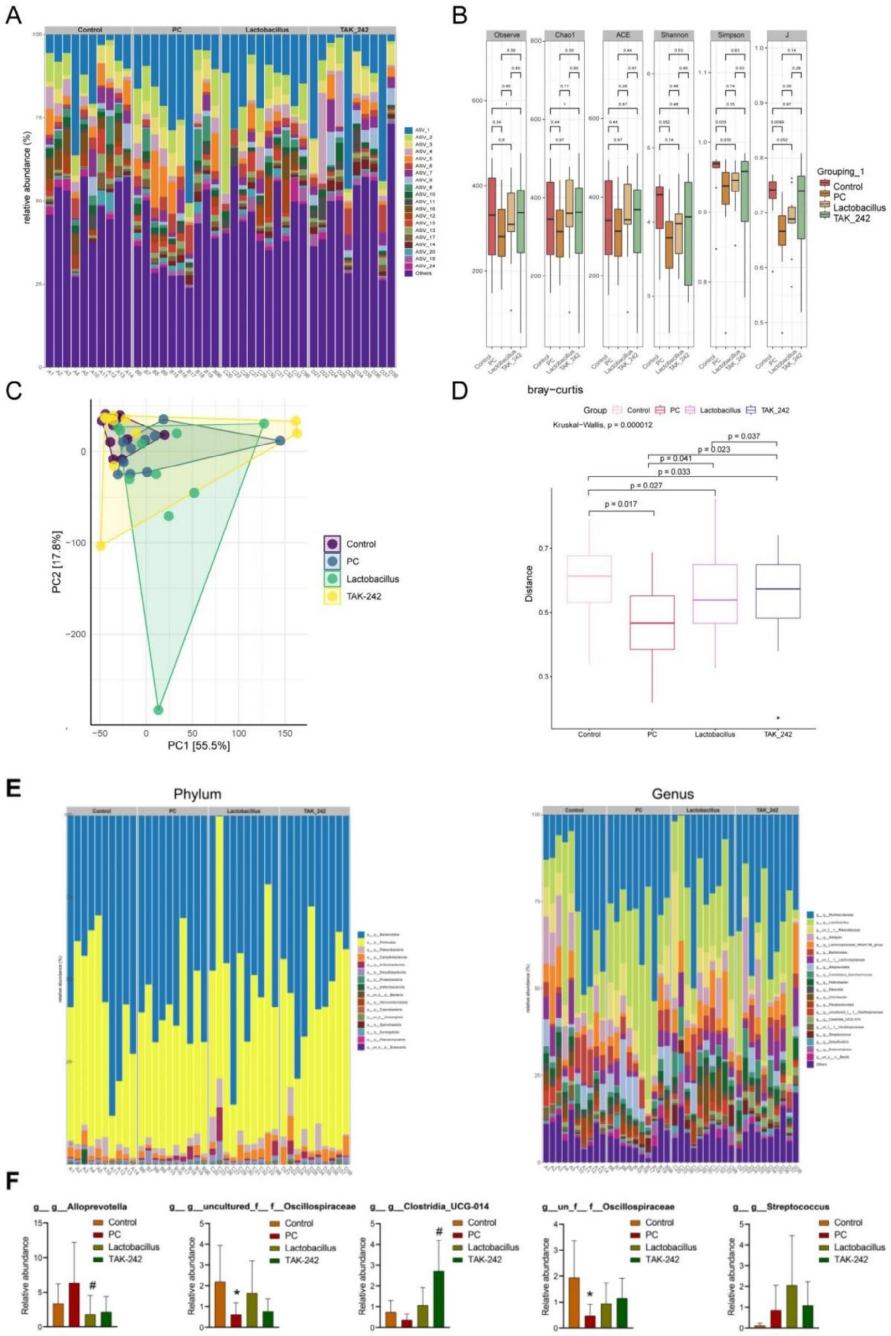
*L. casei* & *L. reuteri* regulates gut microbial homeostasis by inhibiting TLR4. (**A**) ASV analysis of the relative abundance of species. (**B**) Alpha diversity of gut microbiota. (**C**) PCoA analysis of gut microbiota. (**D**) Beta diversity of gut microbiota. (**E**) The relative abundance of gut microbiota at phylum (left) and genus (right) level. (**F**) Differences of relative abundance of specific gut microbiota at genus level. *p < 0.05 versus control group, #*p* < 0.05 versus PC group

Next, we aimed to determine whether and how *L. casei* & *L. reuteri* regulate the gut microbiota of pancreatic tumor-bearing mice. The relative abundance of species was shown in Fig. 4A after amplicon sequence variant (ASV) analysis. Alpha diversity of gut microbiota in pancreatic tumor-bearing mice showed increased tendency after *L. casei* & *L. reuteri* or TAK-242 treatment, but without statistical significance (Fig. 4B). Principle coordinates analysis (PCoA) exhibited that the gut microorganism structures were different between different groups (Fig. 4C). Besides, the beta diversity of gut microbiota in nude mice was enhanced upon *L. casei* & *L. reuteri* or TAK-242 treatment (Fig. 4D). Furthermore, substantial differences were observed between groups at the phylum and genus levels (Fig. 4E). Specifically, *L. casei* & *L. reuteri* reduced *Alloprevotella*, and TAK-242 increased *Clostridia UCG-014* in the gut of nude mice (Fig. 4F). After *L. casei* & *L. reuteri* or TAK-242 treatment, the abundance of *Oscillospiraceae* and *Streptococcus* showed an increased tendency in the gut but exhibited no statistical difference unfortunately (Fig. 4F). These data demonstrated that *L. casei* & *L. reuteri* regulates gut microbial homeostasis by inhibiting TLR4.

### ***L. casei*** & ***L. reuteri*** regulates gut metabolic homeostasis by inhibiting TLR4

**Fig. 5:**
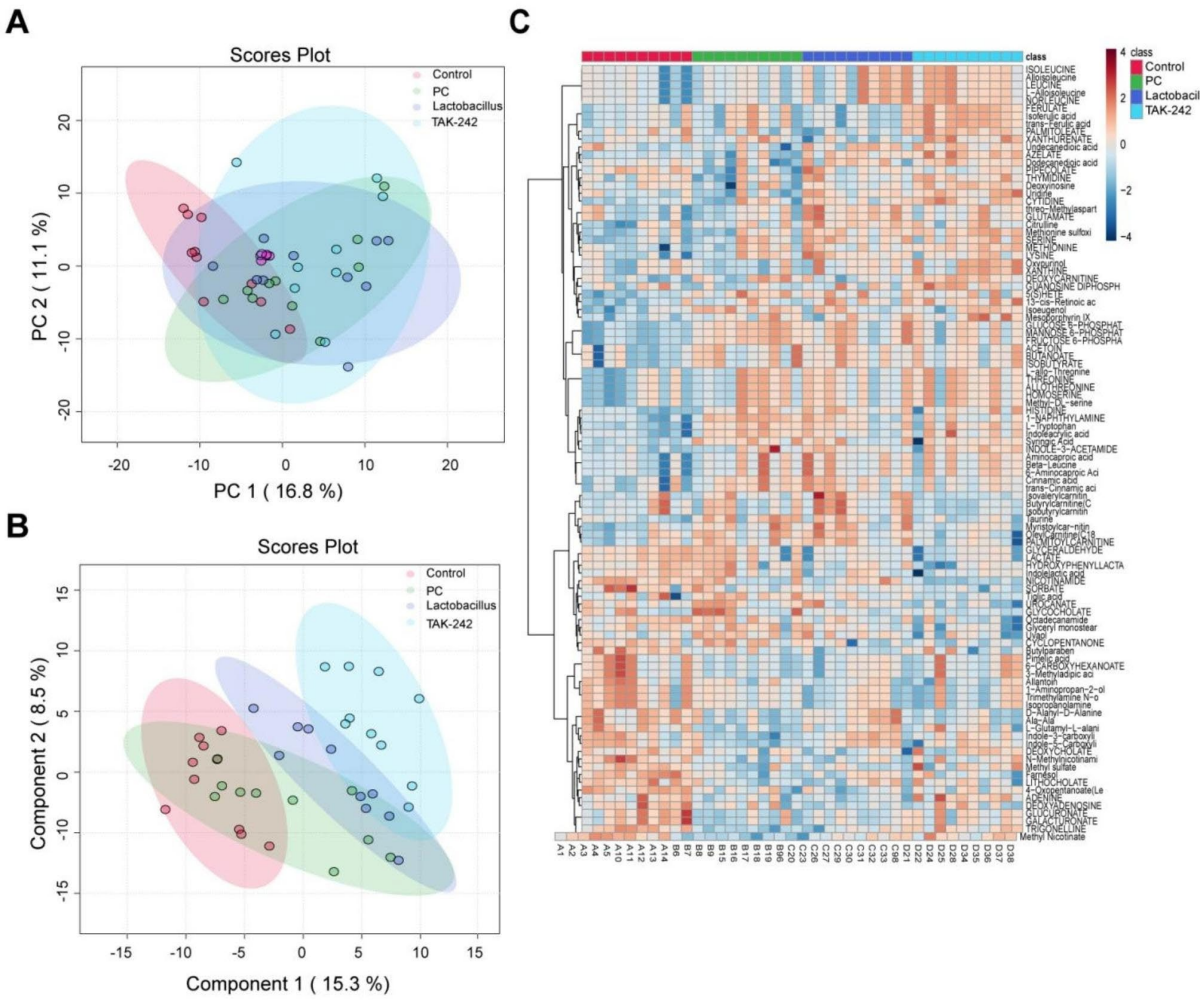
*L. casei* & *L. reuteri* regulates gut microbial homeostasis by inhibiting TLR4. (**A**) PCA scores plot for metabolic profiles. (**B**) PLS-DA plot for metabolic profiles. (**C**) The heat map of the metabolic features

**Fig. 6:**
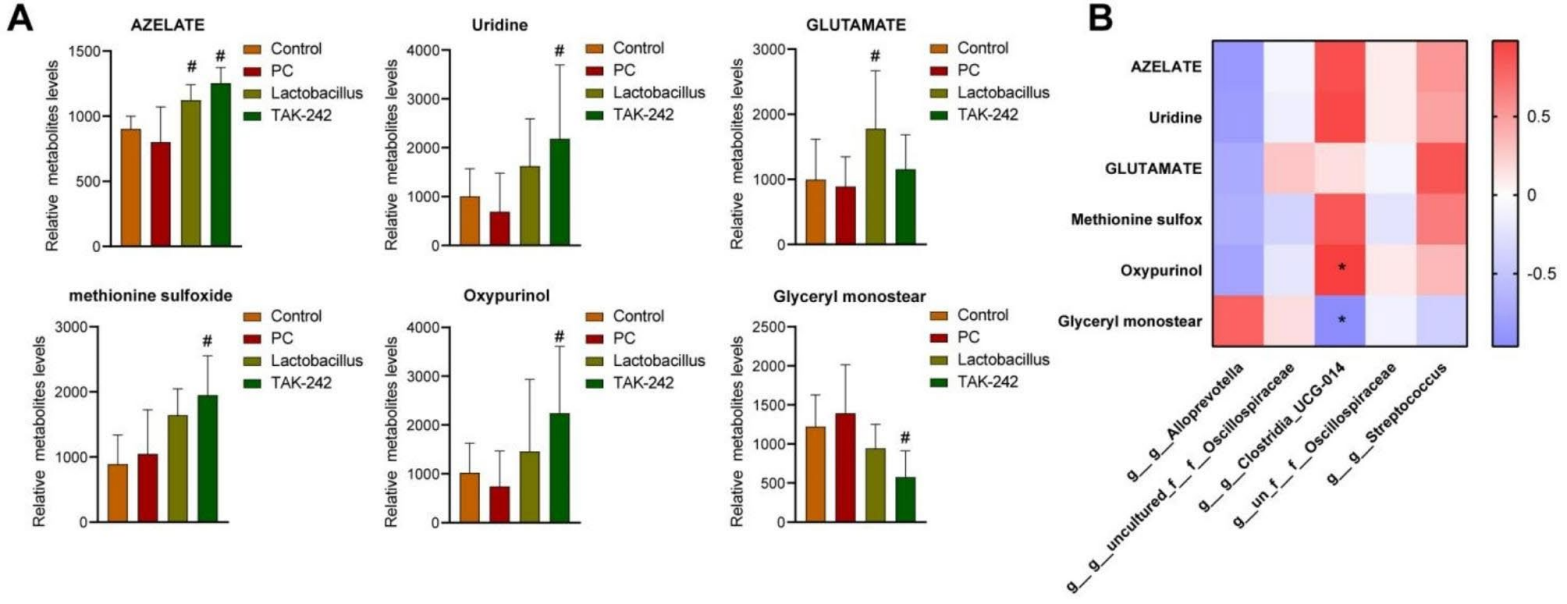
The correlation between gut microbiota and microbial metabolites. (**A**) Differences of relative abundance of specific microbial metabolites. (**B**) Pearson analysis of gut microbiota and microbial metabolites. *p < 0.05

Finally, we collected the feces of tumor-bearing mice and assessed the metabolites using UPLC-MS untargeted metabolomics. The metabolic profiles among groups were quite different as proved by principal component analysis (PCA) (Fig. 5A) and partial least squares-discriminate analysis (PLS-DA) (Fig. 5B). As visually shown by heatmap (Fig. 5C), feces metabolite profiles altered significantly in pancreatic tumor-bearing mice compared with normal mice or mice receiving *L. casei* & *L. reuteri* or TAK-242. Specially, *L. casei* & *L. reuteri* increased fecal azelate and glutamate. TAK-242 increased azelate, uridine, methionine sulfoxide, oxypurinol, and decreased glyceryl monoester in the feces of pancreatic tumor-bearing mice (Fig. 6A). Besides, Pearson correlation analysis showed that fecal oxypurinol and glyceryl monoester levels were positively or negatively associated with gut Clostridia UCG-014 abundance, respectively (Fig. 6B). These results indicated that *L. casei* & *L. reuteri* regulates gut microbial homeostasis by inhibiting TLR4.

## Discussion

Pancreatic cancer is a malignant tumor with extremely poor outcomes after surgery, chemotherapy, radiotherapy, or immunotherapy, so it is of great importance to developing new and effective treatments.

Probiotics, as live microorganisms beneficial to the host, have been widely used in the food and pharmaceutical industry. Recent attempts to use probiotics in the oncology field have pioneered another new direction for the utilization of probiotics and brought new perspectives for tumor prevention and treatment [20, 28]. *L. casei* and *L. reuteri* are LAB strains with probiotic properties, and their roles in a variety of cancers have been reported by several studies. In vitro, *L. casei* promoted the expressions of pro-apoptotic genes and inhibited the expressions of anti-apoptotic genes in cervical cancer cells [29]. *L. reuteri* was able to suppress the proliferation of gastric cancer cells [30]. In vivo, Jacouton et al. found that *L. casei* decreased histological scores and proliferation indices in colorectal cancer mice and inhibited colorectal cancer progression through immunomodulatory and antiproliferative effects [26]. Hu et al. reported that supplementation with *L. reuteri* increased acetate production, and suppressed IL-17 A expression and hepatocellular carcinoma development [25]. Exogenous *L. reuteri* showed inhibitory effects on the growth of colorectal cancer [27]. *L. casei* inhibited the growth of pancreatic cancer cells [31] and increased the therapeutic effect of gemcitabine on pancreatic cancer [32]. However, the therapeutic potential of the combination of these two probiotics on pancreatic cancer is not clear previously. Our results revealed that the combination of *L. casei* and *L. reuteri* inhibited the proliferation, migration, and invasion of pancreatic cancer cells, as well as tumor growth in pancreatic cancer-bearing mice. Therefore, *L. casei* & *L. reuteri* might be a potential approach for the treatment of pancreatic cancer.

TLR4 is a key receptor involved in LPS recognition and LPS signal initiation. After binding LPS, TLR4 is activated and subsequently recruits adaptor molecules, such as MyD88 and Toll/IL-1R domain-containing adaptor-inducing IFN-beta (TRIF), to form downstream signalling cascades. Transcription factors such as NF-κB, activator protein-1 (AP-1), and interferon regulating factor 3 (IRF3) are then activated, resulting in the expression of multiple inflammatory cytokines and interferons [33]. As a double-edged sword, TLR4 exerted pro-tumor or anti-tumor effects in different cancers [34], while in pancreatic cancer, TLR4 mainly promoted tumor progression. TLR4 was significantly overexpressed in pancreatic cancer cells and tissues [35] and boosted the proliferation of pancreatic cancer cells by upregulating anti-apoptotic Bcl-2 [35]. TLR4 facilitated vascular endothelial growth factor (VEGF) expression through activation of PI3K/AKT signalling pathway, which ultimately promotes pancreatic cancer angiogenesis [36]. Besides, TLR4 is also involved in the immune escape of pancreatic cancer. Mechanistically, TLR4 enhanced the expression of the immune checkpoint molecules such as programmed death ligand-1 (PD-L1) [37] and V-domain Ig suppressor of T cell activation (VISTA) [38] in pancreatic cancer cells, as well as upregulated the production of anti-inflammatory IL-35 from regulatory B cells [39], all of which promoted the formation of an immunosuppressive microenvironment in pancreatic cancer. Interestingly, in the present study we found that *L. casei* & *L. reuteri* inhibited the expression of TLR4 and MyD88 in pancreatic cancer cells, and TLR4 inhibitors displayed similar anti-pancreatic cancer effects as *L. casei* & *L. reuteri*, which suggested that *L. casei* & *L. reuteri* exerted their suppressive effects on pancreatic cancer by inhibiting TLR4.

Macrophages are the most abundant immune cells in the TME, and their M1/M2 polarization influences the processes of tumor proliferation, invasion, metastasis, angiogenesis, lymphangiogenesis, and immune escape [40]. Macrophages possess high plasticity and heterogeneity, and M1 or M2 phenotypes are not their end states. Various factors in the TME, including cytokines and exosomes, regulate macrophage polarization to converse between M1 and M2 [6]. Pancreatic cancer cells promoted M2 macrophage polarization in the TME [41, 42], and our results showed that *L. casei* & *L. reuteri* treatment ablated the pro-M2 effect of pancreatic cancer cells on macrophages by inhibiting TLR4. Previous studies have confirmed that TLR4 promoted IL-10 expression [43], and IL-10 facilitated macrophage polarization toward the M2 phenotype [40]. Therefore, we speculated that TLR4 inhibition by *L. casei* & *L. reuteri* in pancreatic cancer cells led to a decrease in IL-10 secretion, which in turn drove M1 macrophage polarization in TME. In addition, we found that *L. casei* & *L. reuteri* altered the gut microbial composition and metabolite productions in pancreatic cancer xenograft mice. Studies have proven that gut microbiota dysbiosis could promote the production of pro-oncogenic metabolites and induce sustained inflammatory responses, oxidative stress, and immunosuppression [17]. More importantly, since the pancreas is anatomically connected to the duodenum through the pancreatic duct, imbalanced gut microbiota might enter the pancreas through the pancreatic duct and alter the pancreatic microenvironment [44]. The aforementioned factors together contributed to the initiation and progression of pancreatic cancer. Therefore, *L. casei* & *L. reuteri* may inhibit pancreatic cancer development by regulating gut microbial homeostasis.

Our study found that L. casei & *L. reuteri* reduced the abundance of *Alloprevotella* in the gut of pancreatic cancer-bearing mice, while increasing fecal azelate and glutamate content. Previous studies showed that *Alloprevotella* was significantly enriched in oral squamous cell carcinoma tissues [45] and *Alloprevotella* was more in outer tumor tissues of oral cancer [46]. Jiao et al. found that compared to normal tissues, *Alloprevotella* was significantly increased in thyroid cancer tissues [47]. Wang et al. on the other hand, observed significant *Alloprevotella* enrichment in the intestinal mucosa of azoxymethane and dextran sulfate sodium-induced model of ulcerative colitis carcinogenesis [48]. These results suggest that *Alloprevotella* might promote tumor progression, but the molecular mechanisms by which *Alloprevotella* regulated tumor progression were not yet clear. Azelate has an inhibitory effect on tumor cell proliferation [49], but glutamate can promote tumor progression [50]. Based on these studies, we speculated that *L. casei* & *L. reuteri* might inhibit pancreatic cancer progression by decreasing intestinal *Alloprevotella* abundance and increasing azelate. Further studies are needed to confirm our speculation in the future.

It is worth noting that although probiotics have shown good efficacy in preclinical studies in pancreatic cancer, these studies included limited and heterogeneous trial samples. Besides, there have been no large randomized controlled trials determining the anti-pancreatic cancer efficacy of probiotics until now. In addition, chronic pancreatitis is a risk factor for pancreatic cancer [51], and there is evidence that probiotics can improve acute pancreatitis [52]. But the potential role of probiotics in improving chronic pancreatitis is still weak, so attempts to prevent pancreatic cancer by alleviating pancreatitis with probiotics do not appear to be reliable.

In conclusion, our results confirmed the efficacy of *L. casei* & *L. reuteri* in pancreatic cancer treatment. Mechanistically, *L. casei* & *L. reuteri* inhibited TLR4 expression in pancreatic cancer cells, which ultimately promoted M1 polarization of macrophages in the TME; Meanwhile, *L. casei* & *L. reuteri* regulated gut microbial homeostasis and metabolite production in pancreatic cancer-bearing mice. This study suggested that *L. casei* combined with *L. reuteri* might serve as a potential therapeutic approach for pancreatic cancer.

### Electronic supplementary material

Below is the link to the electronic supplementary material.

Supplementary Material 1

Supplementary Material 2

## Data Availability

All data found in this study are included in the manuscript or are available upon request by contact with the first author or corresponding author.
