# Supplementary figures and images for: Lactobacillus casei combined with Lactobacillus reuteri alleviate pancreatic cancer by inhibiting TLR4 to promote macrophage M1 polarization and regulate gut microbial homeostasis
Source: BMC Cancer. 2023 Oct 30;23:1044. doi: 10.1186/s12885-023-11557-z (PMC10614400; doi:10.1186/s12885-023-11557-z)

iNOS (110-130KDa)

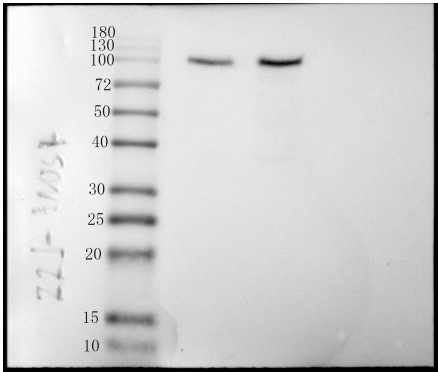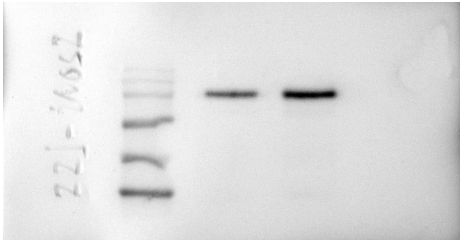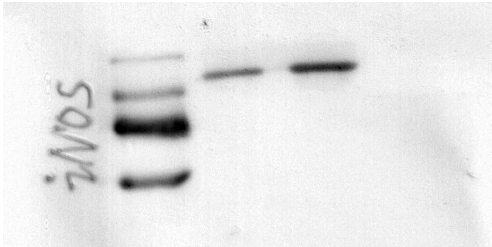

Arg-1 (35-36KDa)

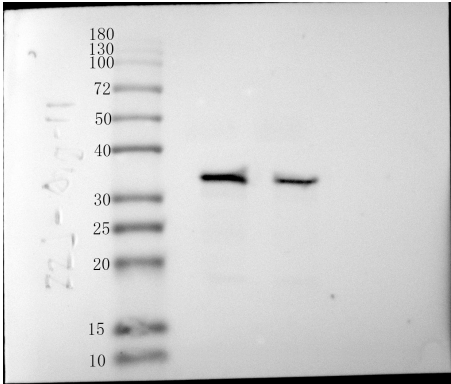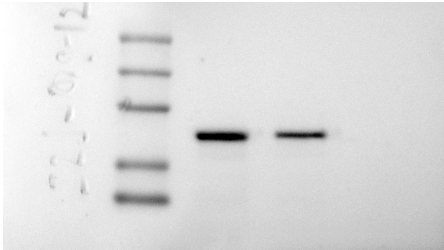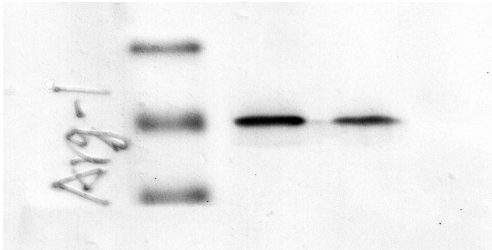

$\beta$ -actin (42KDa)

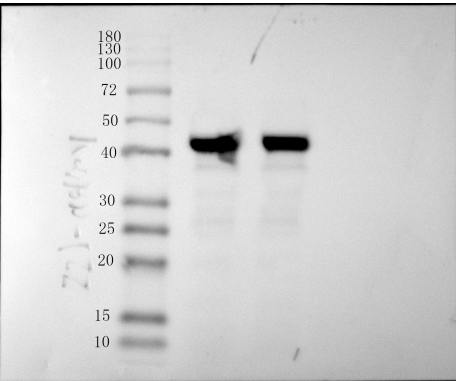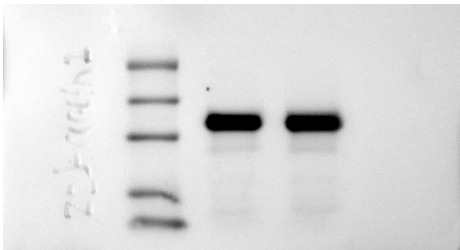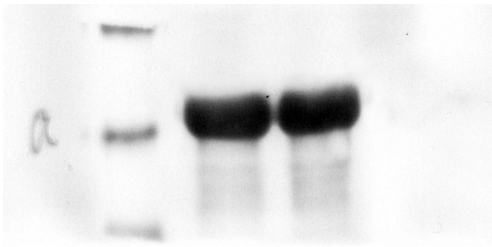

Supplement: Supplementary file 1 — Supplementary Material 1 [file 12885_2023_11557_MOESM1_ESM.pdf]

iNOS (110-130KDa)

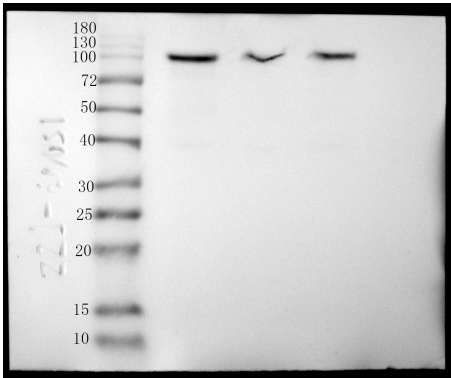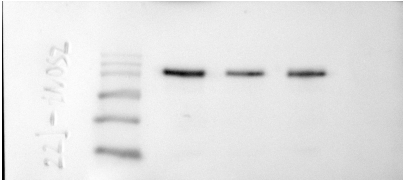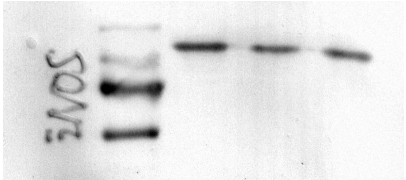

Arg-1 (35-36KDa)

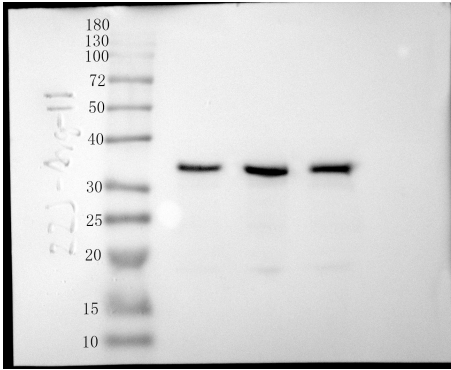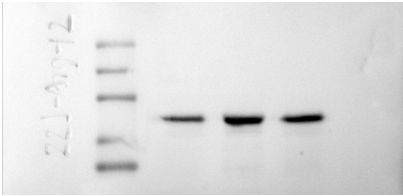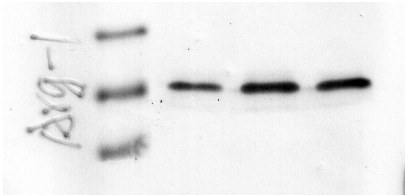

$\beta$ -actin (42KDa)

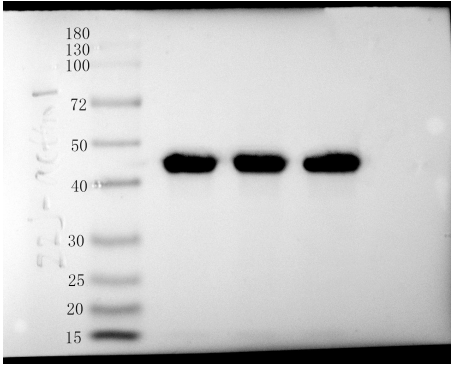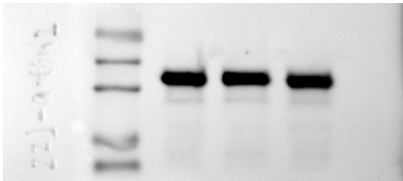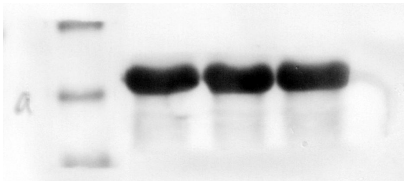

Supplement: Supplementary file 2 — Supplementary Material 2 [file 12885_2023_11557_MOESM2_ESM.pdf]
